# Chronic conditions and multimorbidity in a primary care population: a study in the Swiss Sentinel Surveillance Network (*Sentinella*)

**DOI:** 10.1007/s00038-018-1114-6

**Published:** 2018-05-21

**Authors:** Markus Gnädinger, Lilli Herzig, Alessandro Ceschi, Dieter Conen, Alfred Staehelin, Marco Zoller, Milo A. Puhan

**Affiliations:** 1 Institute of Primary Care, University of Zurich, Zurich, Switzerland; 2 Institute of Family Medicine, University of Lausanne, Lausanne, Switzerland; 3 Swiss Sentinel Surveillance Network (Sentinella), Swiss Federal Office of Public Health, Bern, Switzerland; 4 Division of Clinical Pharmacology and Toxicology, Institute of Pharmacological Sciences of Southern Switzerland, Ente Ospedaliero Cantonale, Lugano, Switzerland; 5 Department of Clinical Pharmacology and Toxicology, University Hospital Zurich, Zurich, Switzerland; 6 Patientensicherheit Schweiz, Zurich, Switzerland; 7 Epidemiology, Biostatistics, and Prevention Institute, University of Zurich, Zurich, Switzerland

**Keywords:** Morbidity, Drug treatment, Care dependency, Hospitalization, Primary health care, Switzerland

## Abstract

**Objectives:**

To provide estimates of the prevalence of chronic conditions in Swiss primary care.

**Methods:**

In total, 175 general practitioners (GP) or pediatricians (PED) reporting to the Swiss Sentinel Surveillance Network collected morbidity data.

**Results:**

In 26,853 patient contacts, mean (± SD) age was 55.8 ± 21.6 or 6.1 ± 5.7 years (in GPs vs. PEDs, respectively) and 47% were males. In GP patients, median Thurgau Morbidity Index was 2 (IQR 1–3). The median numbers of chronic conditions and permanently used prescribed drugs were 2 (0–5) and 2 (1–4), respectively; in PEDs medians were 0. Out of all patients, 16.7 and 7.0% of the PED patients were hospitalized during the previous year; patients cared by family/proxies or community nurses were hospitalized significantly more often than patients living in homes (50.1 vs. 35.4%, OR 1.41, *p* < 0.001). Out of patients over 80 years of age, 51.5% were care dependent and 45.5% of the patients over 90 years were living in homes for the elderly.

**Conclusions:**

In a representative sample of Swiss primary care patients, a substantial part shows multimorbidity with a high prevalence of chronic diseases, multiple drug treatment, and care dependency. These data may serve to be compared with other patient groups or other primary care systems.

*Trial registration* www.clinicaltrials.gov NCT0229537, national study registry www.kofam.ch SNCTP000001207.

**Electronic supplementary material:**

The online version of this article (10.1007/s00038-018-1114-6) contains supplementary material, which is available to authorized users.

## Introduction

Due to the aging of most societies worldwide, there has been an increase in the prevalence of chronic conditions and multimorbidity (Uijen and van de Lisdonk 2008; Barnett et al. 2012; Prados-Torres et al. 2014). We define multimorbidity as three or more conditions that accumulate in one subject, irrespective of whether these conditions are related or not. This needs to be distinguished from comorbidities, which refer to conditions related to disorders of primary interest. For example, renal failure, peripheral neuropathy and retinopathy are comorbidities of diabetes and thus form a cluster of interdependent conditions (Prados-Torres et al. 2014).

Most of the elderly people with multiple chronic conditions are cared for in primary care. However, there are concerns that the system cannot keep pace with the chronic disease epidemic leading to an inappropriate provision of care (Bodenheimer et al. 2009). At present, persons with multimorbidity need to devote a substantial amount of time for health care visits and disease management. Due to functional losses and pain, they have to take various medicines that increase patients’ risk for polypharmacy or drug interactions (Blozik et al. 2013). Moreover, patients with multiple chronic conditions are more likely to become socially isolated, to experience financial difficulties as well as a loss of life years (Gijsen et al. 2001). The patients’ families and proxies may be involved as informal care givers and deliver care which poses additional challenges in terms of time and required resources (Tennstedt et al. 1989; Häusler et al. 2017). Eventually, multimorbidity also affects the society with regard to higher costs, the need to provide healthcare facilities and a decrease of patient’s workforce (Federal Office of Public Health 2015).

Data on the burden of chronic diseases are important to plan for appropriate health care services for patients with chronic conditions and multimorbidity in Switzerland and internationally. However, valid and nationally representative epidemiologic data about multimorbidity are often scarce in Switzerland and other countries. In terms of Swiss inpatient services, there is a solid data basis, whereas data on outpatient services including primary care where patients with chronic conditions are primarily cared for are lacking.

### Aims

The aim of this study was, therefore, to provide valid and representative epidemiologic estimates of the prevalence of chronic conditions and multimorbidity in the Swiss primary care population.

## Methods

### Sample

This analysis was based on the data generated by the Swiss Sentinel Surveillance Network (*Sentinella*) (Gnädinger et al. 2015). *Sentinella* is a network of approximately 180 general practitioners (GPs) and pediatricians (PEDs) and was founded in 1986 in order to survey communicable diseases in Switzerland (Federal Office of Public Health 2018). Later, other issues related to family medicine were also investigated in this system. Furthermore, it performs a denominator analysis twice a year to define its patient collective. For the present study, the analysis of physician-to-patient contacts (PPC) was expanded by the collection of data related to multimorbidity. Each patient who was consulting a GP or PED practice participating in *Sentinella* between March 7th and March 20th, 2015 was included in the statistical analysis (patients consulting twice, or more were included for each visit). Patients refusing data transmission to the *Sentinella* system were excluded from the analysis. Furthermore, to characterize their practices and to evaluate potential difficulties with the study methodology, the *Sentinella* physicians completed two questionnaires, one at the beginning and one at the end of the study (Gnädinger et al. 2017).

Written instructions were delivered to the participating physicians by the *Sentinella* administration (Appendix A in Electronic Supplementary Material). Detailed information on the definitions of the study parameters given to the physicians is shown in Appendix B in Electronic Supplementary Material. Appendix C in Electronic Supplementary Material lists frequently asked questions.

### Questionnaires

The year of birth and gender were recorded for each patient. Physicians provided the Thurgau Morbidity Index (TMI) (Fischer et al. 2007) as the primary indicator for the prevalence and degree of severity of chronic conditions and multimorbidity; the TMI increases with the number of chronic conditions as well as their severity (a detailed description of the TMI can be found in the Appendix B in Electronic Supplementary Material). As secondary indicators, we included the number of chronic conditions (irrespective of their severity), the number of prescribed drugs taken regularly, the Evans’ Index (co-morbidity polypharmacy score) (Evans et al. 2012), any hospitalization during the previous twelve months, and care dependency. The Evans’ Index was calculated by the simple addition of the numbers of chronic conditions and drugs taken regularly. Since the physicians completing the questionnaires were not trained in using a detailed nursing scale to measure care dependency (Noelker and Browdie 2014), we created a simple four-point Likert-type scale item (i.e., no care, care by proxies, by community nurse (CNS), by an institution); because usually all parents give care to their children, so logistic regression analysis was restricted to adult patients > 20 years. A follow-up visit was defined as a second or further visit during the fourteen days of data collection (we could not differentiate between no follow-up visit or a missing answer since physicians only reported if a repeat visit occurred).

### Assessment of how the study sample represented Swiss GPs and the target population

For each physician, we assigned the *Sentinella* coding number and asked for the specialization as well as the language. To determine the representativeness of our sample, we performed some comparisons: firstly, we compared our records to the data obtained by the NewIndex AG, Olten (a merger of Swiss trust center organizations excluding the canton of Vaud) for 2014. Most physicians are contracted to a trust center; therefore, the data should reflect a representative sample of Swiss physicians. Secondly, we compared the *Sentinella* physician characteristics (age, gender, specialization) with the dataset of 2014 obtained from the Swiss Medical Association (FMH) in Berne which includes all Swiss physicians with information on their specialization. Virtually all physicians working in Switzerland are members of the FMH. Thirdly, to verify complete inclusion, we compared our data with those from an earlier *Sentinella* fourteen-day analysis (August 2014) that was limited to the collection of gender and age data. And finally, to describe the practice size, we received the number of PPC for 2015 from the *Sentinella* administration. Where applicable, our publication follows the general STROBE guidelines (Equator, the Network 2018) (Appendix D in Electronic Supplementary Material).

### Statistical methods

Values are given as frequencies, mean ± SD or median [interquartile range (IQR)], depending on the distribution of the data. Medians were approximated by Hampel. TMI scales were compared with Wilcoxon rank sum test.

As the numbers of drugs and conditions as well as the ordinal data level of TMI or care-dependency variables were not normally distributed, correlation analyses were performed with Spearman’s Rho. To assess the representativeness of the patients and participating physicians, we used unpaired *T*- or Chi square tests to identify statistically significant inferences.

To assess the association of multimorbidity with hospitalization, we used the SPSS GENLINMIXED procedure, a procedure that fits generalized linear mixed models. Clustering of patients was addressed using a mixed binary logistic regression with the fixed factors of gender, age, care dependency, number of chronic drug treatments, number of chronic conditions, and TMI as well as the physician’s practice number as a random factor. If one item was missing, the whole record was excluded from the analysis. For the statistical analyses, we used the software SPSS 24 (IBM SPSS 2018).

## Results

### Records

We received 26,853 PPC data records; 27.5% were transmitted electronically and the remaining files as hard copies by mail. In total, 22,379 records focused on weeks 11 and 12, 2504 on week 13 and 1970 records on weeks 8–10 and 14–26, respectively.

### Description of study physicians and comparison to all Swiss GPs and PEDs

During 2015, 151 practices were registered in the *Sentinella* system (where a physician’s code does not necessarily correspond to one physician only), corresponding to 193 physicians. Out of the 151 practices, 144 (94.7%) which corresponds to 180 physicians regularly reported to the *Sentinella* system (which means that they announced PPCs for at least 39 weeks a year). In total, 119 practices comprised one reporting physician, 19 two, 5 three, and 1 eight, adding up to 180 physicians. From all physicians, 122 (67.8%) were German, 44 French (24.4%) and 14 Italian speaking (7.8%). Further characteristics are listed in Table 1 which also provides comparative information with FMH data on all Swiss physicians. For the statistical analyses, two practices (5 GPs) regularly to the *Sentinella* system were excluded as they did not participate this denominator analysis. This led to a study sample of 142 practices and 175 physicians.

**Table 1:** Comparison of the *Sentinella* versus Swiss Medical Association (FMH) physician collectives (MIPC study, Switzerland 2015)

Parameter	*Sentinella* collective 2015^a^	FMH collective 2014
Number of physicians	180	6929
Gender		
Male	71%	66%
Female	29%	34%
Age categories		
< 40 years	7%	9%
40–49 years	24%	25%
50–59 years	37%	34%
60 years and older	32%	32%
Specialty		
GP	82%	86%
PED	18%	14%

^a^Comparisons of *Sentinella* and Swiss Medical Association (FMH) groups by Chi square were not significant. The two practices that did not report morbidity data were also included in this table, because they were otherwise part of the *Sentinella* physician collective

### Response rate and difficulties in variable coding

During 2015, the mean of PPC was 4456 ± 2137 for GPs, and 5297 ± 2715 for PEDs. Figure e1 (Appendix E in Electronic Supplementary Material) summarizes the response rates of the different questionnaire items. Out of 20,799 records concerning adult patients (> 19 year), all variables (age, gender, number of conditions and drugs, TMI, care dependency, and previous hospitalization) were coded in 18,297 cases (88.0%). As a measure of completeness of reporting 2 weeks of morbidity data, we assumed that a proportion of 3.3% or more of the yearly PPCs would be submitted to our study database; this was achieved by 161 (92.0%) of the physicians. Items concerning problems of the study physicians with coding of the morbidity variables are listed in Table e1 (Appendix E in Electronic Supplementary Material) and the frequently asked questions in Appendix C in Electronic Supplementary Material.

### Description of patients and comparison to all patients in Swiss primary care

Out of the 26,853 records, 12,606 could be allocated to male patients (47.0%), 14,209 to females (52.9%), whereas for 38 (0.1%) cases information on gender was missing. Table e2 (Appendix E in Electronic Supplementary Material) summarizes the age categories separated by gender and compares them with the New Index data for GPs; Table e3 (Appendix E in Electronic Supplementary Material) lists the same information for PEDs. This comparison demonstrates that the patients consulting the *Sentinella* physicians are representative of the overall Swiss primary care population. A comparison of a fourteen-day analysis of age and gender from August 2014 with the current data did not reveal any significant differences of age (47.2 ± 27.5 vs. 47.5 ± 27.1 years) and gender (47.0 vs. 47.5% males) proportions (2015 vs. 2014, respectively).

### Prevalence of chronic disease, multimorbidity, and polypharmacy

**Fig. 1:**
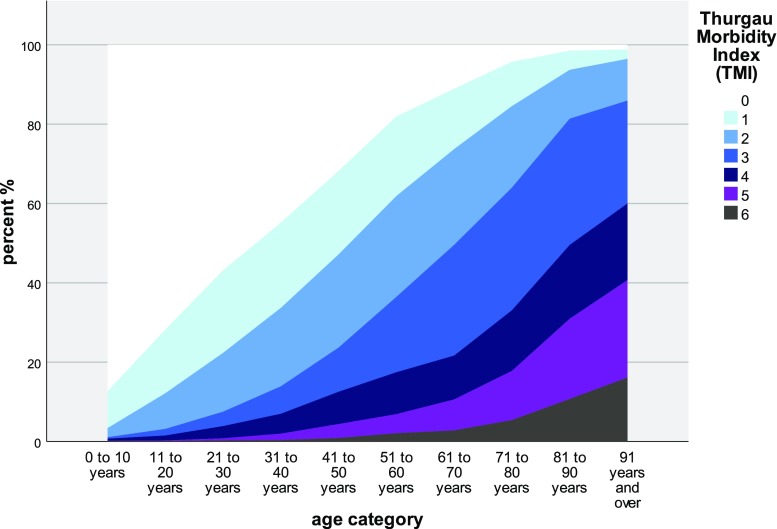
Thurgau Morbidity Index* (TMI) values, percent % (MIPC study, Switzerland 2015). *The index values denote: “0” healthy, “1” premorbid, “2” one or two mild-to-moderate conditions, “3” three and more mild-to-moderate conditions, “4” one severe and less than three mild-to-moderate conditions, “5” one severe and three or more mild-to-moderate conditions, “6” two or more severe conditions. Graduations denote the entire class

TMI scale values in GP practices were: 0 in 4752 patients (23.7%), 1 in 3160 (15.7%), 2 in 3972 (19.8%), 3 in 3854 (19.2%), 4 in 2099 (10.5%), 5 in 1537 (7.5%) and 6 in 702 (3.7%) (20,076 valid and 1876 missing recordings). In PEDs, the results were: 0 in 3711 (85.4%), 1 in 451 (10.4%), 2 in 130 (3.0%), 3 in 23 (0.5%), 4 in 20 (0.5%), 5 in 2 (0.0%), and 6 in 7 (0.2%) patients, respectively (4344 valid and 557 missing recordings). The distribution of TMI data by age group is shown in Fig. 1. Among the four care-dependency groups (none, proxies, CNS, nursing home), TMI values were 1.6, 4.0, 4.1, and 4.3 as estimated by Hampel. The CNS vs. nursing home group values were not significantly different (Wilcoxon).

**Fig. 2:**
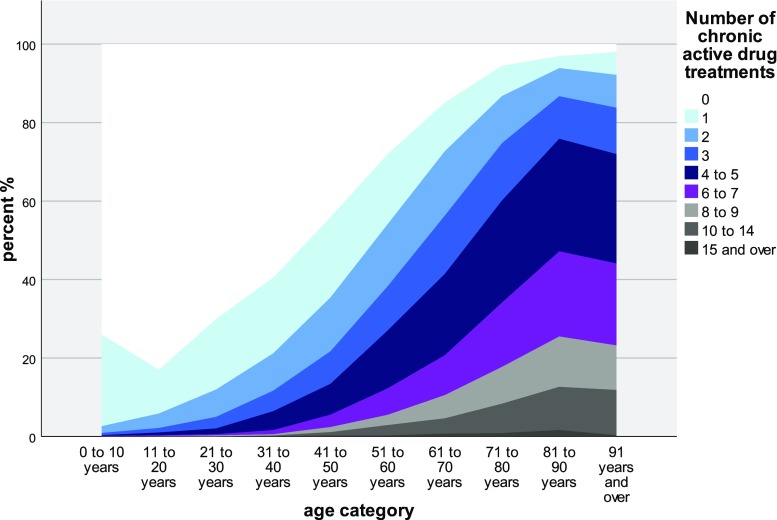
Number of prescribed drugs* regularly taken, percent values (%) (MIPC study, Switzerland 2015). *Graduations denote the entire class

The secondary indicators of multimorbidity showed a similar pattern. Table e4 (Appendix E in Electronic Supplementary Material) summarizes the morbidity variables (hospitalization, care dependency, condition and drug counts, Evans’ Index, TMI, and follow-up visit) by age categories and gender. The number of *chronic conditions* in GP patients was 2 (1–4) (median, interquartile range [IQR]) and in PEDs 0 (0–0). The spread of chronic conditions is depicted in Figure e2 (Appendix E in Electronic Supplementary Material). In GP patients, the median number of prescribed *drugs* taken regularly was 2 (0–5) and in PEDs 0 (0–0); the maximum number of regularly taken drugs was 25 in GPs and 7 in PEDs. Polymedication (> 4 drugs) was present in 20.7% of the patients, increasing to 60.9% in very elderly (80+) individuals. The distribution of the number of chronically taken drugs by age is depicted in Fig. 2. The median value of the *Evans Index* was 4 (1–9) in GPs and 0 (0–1) in PEDs; the age distribution is depicted in Figure e3 (Appendix E in Electronic Supplementary Material).

*Hospitalization* during the previous year was reported in 3383 of 20,280 records (16.7%) in GPs (1672 missing), and in 315 of 4481 records (7.0%) in PEDs (420 missing). Logistic regression (GENLINMIXED procedure) showed positive and statistically significant associations of hospitalization with care dependency, age, number of chronic drug treatments, number of chronic conditions, and TMI; females had a weak but not statistically significant negative association with previous hospitalization (Table 2). The model showed a negative predictive value of 96.6%, and positive predictive value of 24.9% for previous hospitalization. Outpatients (care-dependency grades 1 and 2) were statistically significantly more frequently hospitalized than inpatients living in homes (grade 3) (50.1 vs. 35.4%, OR 1.41, *p* < 0.001 by Chi square test); this association remained statistically significant in the adjusted analysis (Table 2).

**Table 2:** Logistic regression using hospitalization in the previous year as target variable (odds ratios of adult patients with complete records) (MIPC study, Switzerland 2015)

	Crude (mean, 95% CI)	Adjusted^c^ (mean, 95% CI)
Gender^a^	0.997 (0.904–1.055), *p* > 0.05	0.908 (0.832–0.991), *p* = 0.030
Age (per year)	1.029 (1.027–1.032), *p* < 0.001	0.994 (0.991–0.997), *p* < 0.001
Conditions (per naming)	1.294 (1.274–1.315), *p* < 0.001	1.038 (1.015–1.062), *p* = 0.001
Drugs (per naming)	1.244 (1.229–1.259), *p* < 0.001	1.031 (1.011–1.050), *p* = 0.002
Thurgau Morbidity Index (per grade)	1.903 (1.849–1.958), *p* < 0.001	1.650 (1.584–1.718), *p* < 0.001
Care dependency^b^ by		
Family/proxies	6.844 (5.914–7.920), *p* < 0.001	2.875 (2.445–3.380), *p* < 0.001
Community nurse	8.474 (7.196–9.979), *p* < 0.001	3.219 (2.743–3.949), *p* < 0.001
Institution/home	4.297 (3.725–4.958), *p* < 0.001	1.515 (1.284–1.788), *p* < 0.001

^a^(1 = male, 2 = female)

^b^Compared to none, *n* = 18,297

^c^Adjusted for all other variables in the table

*Multiple visits* during the fortnight interval were recorded as follows: in GPs 1703 out of 21,022 PPCs (8.2%, 930 records excluded), and in PEDs 241 out of 4901 (4.9%) PPCs. Because of a misunderstanding, five physicians marked all patients known to the practices as follow-up visits; their records were excluded (see above). In the GP patient group, the mean patient age of records with a second or further visit was one year older compared to those visiting the practice only once (56.9 ± 21.7 vs. 55.8 ± 21.7 years, *p* = 0.042), whereas in PED practices the opposite was the case (5.3 ± 5.6 vs. 6.1 ± 5.7 years, *p* = 0.035). Gender distribution was not significantly different in follow-up compared to first visits (48.0 vs. 46.7% males).

**Fig. 3:**
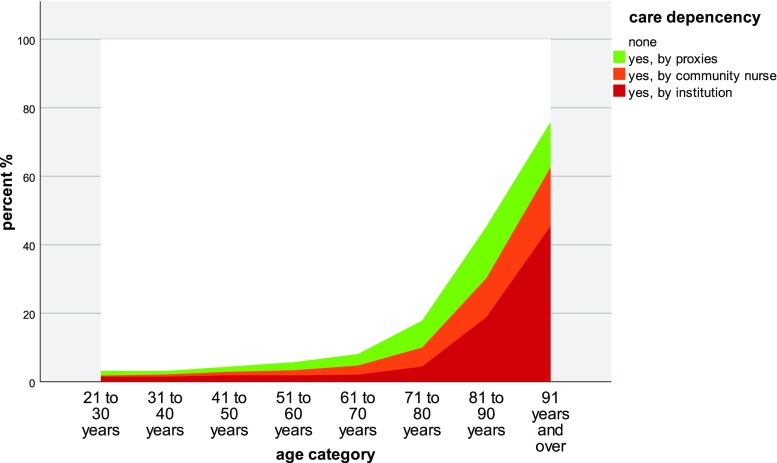
Care dependency* by age groups (percent values %, adult patients only) (MIPC study, Switzerland 2015). *Graduations denote the entire class

Age distribution of *care dependency* is depicted in Fig. 3. Because our questionnaire did not offer the answer “care of minors by parents”, this item was equivocal and could not be evaluated in pediatric patients.

### Correlations among measures of multimorbidity and regional variation

The correlation matrix (Spearman’s Rho) revealed that all variables (previous hospitalization, care dependency, number of prescribed drugs regularly taken, number of chronic diagnoses, TMI and Evans Index) were statistically significantly correlated with each other. They also correlated with age and—except for hospitalization—gender (Table e5, Appendix E in Electronic Supplementary Material). Compared to patients living in German or French speaking regions, a statistically significantly lower morbidity load of patients living in the Italian speaking region was found. This applies to TMI, number of drugs, number of conditions, and Evans’ Index (Figures e4 to e7, Appendix E in Electronic Supplementary Material). However, the sample size was small as expressed by the large error bars.

## Discussion

We collected morbidity data in a primary care setting by the *Sentinella* network with representative physician and patient collectives in Switzerland (participation rate of 90%). In adult patients, we found a median TMI value of 3, which means that half of the patients had at least three or more chronic conditions of mild-to-moderate severity. Similarly, half of the patients had three or more regular drug treatments. Half of the patients over 80 years of age were care dependent.

### Thurgau Morbidity Index and Evans’ Index

In our study, the Thurgau Morbidity as well as the Evans’ indices increased with increasing age. The former index was developed to predict cost in insurance collectives (Fischer et al. 2007). The latter has shown to correlate with survival in trauma patients (Evans et al. 2012; Holmes et al. 2014), in-hospital complications and the need for extended care facilities (Justiniano et al. 2015), and re-admissions (Housley et al. 2015). When coding TMI, the same condition can have different impacts depending on whether the condition is active or inactive, stable or unstable, or socially sensitive or non-sensitive. This aspect solves the problem of diagnosis splitting of list-based indices (e.g., hypertension with or without end-organ damage), but introduces subjectivity to the coding of TMI. On the one hand, TMI does not differentiate between conditions of mild and moderate degree. On the other hand, we did not receive questions from the participating physicians concerning the TMI (see Appendix C in Electronic Supplementary Material). This leads to the conclusion that after some training, TMI coding works easily and intuitively. Although it may be easy to code TMI by the GPs, it may not be appropriate for automated index construction from existing databases. In contrast to an earlier study on Swiss patients by Fischer et al. (2007), we found a shift of TMI codes to the left side, indicating less morbidity (Figure e8, Appendix E in Electronic Supplementary Material); however, that study did not include consecutive patients and was designed to predict insurance costs which, therefore, tended to include a more severely ill patient population (Fischer 2016, personal communication). The lower TMI and morbidity values of patients living in the Italian part of Switzerland (Tessin) cannot be explained by our study group; in the tables provided by the Swiss Federal Statistical Office, there was a slightly higher life expectancy of Tessin compared to Switzerland (males 80.7 vs. 80.2; females 85.4 vs. 84.5 years), a higher mean age when entering to a nursing home (83.0 vs. 81.5 years), and a lower number of nursing home places (< 40.0 vs. 64.5 per 1000 inhabitants) (FSO 2018).

### Number of chronic conditions

**Fig. 4:**
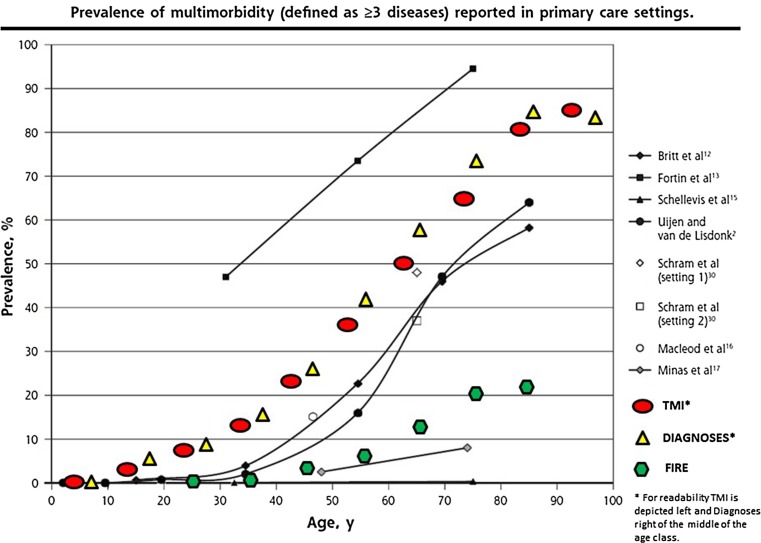
Comparison of Thurgau Morbidity Index (≥ 3) or chronic conditions (≥ 3) with literature^a^ (Fortin et al. 2012, see figure 3 of that article, with permission) (MIPC study, Switzerland 2015). ^a^This review collected consultation-derived information in primary care settings from several studies and compared the prevalence of three or more chronic conditions by age groups. However, the Swiss FIRE data (Rizza et al. 2012) were not consultation- but registry-based

We compared our data on morbidity with those from a review by Fortin et al. (2012) which is suitable for comparisons with earlier European, Canadian and Australian studies (Fig. 4). As expected, within our data set the TMI coding of 3 and higher was slightly less frequent compared to the reporting of three and more chronic conditions, because the conditions count additionally included latent and past diagnoses. The computer-based Swiss data by Rizza et al. (2012) deriving from the FIRE project showed a much lower rate of three and more conditions compared to our study. This difference may be explained by the fact that our data were consultation based, whereas the ones by FIRE were registry based (ill patients have more visits than healthy people). Furthermore, the FIRE physicians showed significant underdiagnosing of common disorders (Zellweger et al. 2014); probably, this was less often the case in our cross-sectional study, in which the physicians extensively reviewed the patient records during a fortnight, whereas the FIRE data reflect every day work that is lacking such detailed review.

An overall population cohort study in the city of Lausanne on self-reported and measured multimorbidity found an overall prevalence of 23–56% depending on the definition used (Pache et al. 2015). In the same Sentinel system as ours, Excoffier et al. (2017) found 35.0 (31.6–38.5) chronic conditions. Guidelines provide recommendations for patients with one single condition. However, multimorbidity is no exception in primary care (Treadwell 2015). Following guidelines developed for each single condition in multimorbid patients may be complicated, time consuming or even dangerous for patients and might also lead to conflicts and more costs (Boyd et al. 2005; Markun et al. 2014). In the same year 2015 Haller et al. investigated the prevalence of multimorbidity in the *Sentinella* patient collective and found a prevalence of 52.1% for ≥ 2 and 35.0% for ≥ 3 chronic conditions, with no significant gender differences (2018). The most prevalent conditions were cardiovascular (42.7%), psychological (28.5%), and metabolic or endocrine disorders (24.1%).

### Number of drugs taken regularly

A Swiss study by a health insurance collective revealed polypharmacy in 17% of the population, increasing to 50% in very elderly (80+ years) (Blozik et al. 2013). These proportions are in line with those of the present study (20.7 and 60.9%, respectively). In contrast to an Italian study by Nobili et al. (2009), who described a mean number of 2.4 ± 2.4 (± SD) prescribed drugs taken regularly by patients aged 65 years and older in 2003, our patients of this age group used, on average 4.9 ± 3.3 drugs. But there were some differences in the definition of regular treatments: in the work by Nobili, the cutoff was 12 months of treatment, whereas in our study it was 1 month. In comparison to our study, Nobili excluded herbal medicines. Furthermore, we included *topical* treatment with possible systemic reactions. The Nobili data were registry based and our data set was collected from PPCs visits. A Swedish publication by Skoog et al. (2014) confirmed our observation that drug prescription increases with age, female gender, and morbidity. In a Belgian cohort study with very elderly (80+ years) individuals, Wauters et al. (2016) described a median number of five regularly used drugs. In this study, the female gender, low education, moderate alcohol consumption, multimorbidity, depression, and a lack of physical activity were linked to polypharmacy. An American study on patients at the time of hospital discharge described an increased risk of polypharmacy (> 16 drugs) in patients with two or more of the following high-risk diagnoses: COPD, cancer, diabetes mellitus, congestive heart failure, and coronary heart disease (Rohrer et al. 2013). The reduction in the proportion of young patients with a single regular treatment from the first to the second decade (24 vs. 18%, Fig. 2) could possibly reflect the vitamin D rickets prophylaxis of 0 to three-year-old infants. We did not evaluate the appropriateness of medication in our study patients. However, another study is now investigating the reduction of inappropriate medication in multimorbid patients (Hasler et al. 2015). A recent Swedish study by Rausch et al. (2017) reported the total number of used drugs and inappropriate medication as associated with hospitalizations for unintentional poisoning.

### Hospitalization

We found that (previous) hospitalization was best predicted by the TMI value, and somewhat less by the care-dependency scale. However, TMI values were not independent of the hospitalization status—a hospital stay can redefine a given condition coding from mild/moderate to severe. Therefore, the correlation observed in our study may reflect an inverse causality, i.e., from the hospitalization to the TMI. Interestingly, institutionalized patients had a lower risk of being hospitalized as compared to people cared by their family or proxies, as well as by the community nurse (OR 6.8 and 8.5 vs. 4.3); this association also remained statistically significant in the adjusted analysis. People cared for by CNS are on risk for adverse drug reactions, medication errors, and hospitalization (Eliot et al. 2016). Although care by CNS may prevent the need for stationary care (Markle-Reid et al. 2006), there might still be an excessive need for it in CNS cared individuals compared to nursing home-dwelling persons. In the case of acute illness, this aspect could be explained by resilient caring networks for institutionalized persons in comparison to people living at home. Another explanation could be that caregivers were more reluctant to hospitalize patients with progressing disease status living in homes because no curative treatment was possible and care could be delivered in the home, as well.

### Care dependency

The current report of the European Observatory on Health Systems and Policies mentions that 4.2% of the Swiss population receive professional long-term care; 64.0% of them are at home and 36.0% in an institution. Additionally, 4.7% of the population (and 16.5% of those over 75 years) receive care by their family or proxies, excluding persons cared for by migrant workers (De Pietro et al. 2015). The Swiss Federal Statistical Office data show that 1.5% of the population of 65 and older lives in a nursing home (FSO 2018). The mean stay lasts for 2.5 years. The mean age of entry to the home is 81.5 years.

Care dependency causes a lot of consequences, such as loss of personal independency, a burden to the social network, and financial demands (Bähler et al. 2015; Jaspers et al. 2015). An ongoing study investigated the disease and treatment burden of Swiss primary care patients (Déruaz-Luyet et al. 2017). In a group of 888 multimorbid patients, they found 5.5+2.2 chronic conditions and 7.7+3.5 prescribed drugs. In our study, half of the patients in the age group over 80 years were care dependent and almost half of the seniors over 90 years lived in homes for the elderly. A substantial proportion of the care was delivered by family and proxies as informal caregivers. In contrast to other countries, in Switzerland more than half of the money spent on care is covered by private expenditures irrespective of whether the care is carried out by professional or informal caregivers (OECD 2011). This seems important as care for inpatients living in homes for the elderly costs six times more than care for outpatients (1.8% of gross domestic product compared to 0.3%, respectively) (OECD 2011). This leads people in need to forego health care services due to financial reasons (Bodenmann et al. 2015).

### Strengths and limitations

The strengths of the study include a Swiss representative sample of physicians and patients, data collection by research-experienced physicians, as well as a large sample size.

The possible weaknesses of our study are that we did not have the opportunity to implement systematic data quality control measures such as double entry or controls within one physician. Furthermore, we did not assess specific chronic conditions but used global measures of multimorbidity. The TMI is not validated as a measure of morbidity and is prone to subjectivity in judgement of chronic condition severity. However, this is a challenge for every method that is used for the assessment of chronic conditions and their severity. Another limitation is that certain drugs taken by the patients but unreported to the physicians could not be recorded. The fortnight study period (March) cannot reflect seasonal changes (e.g., flu epidemic) so that the results may be different over the course of a year.

### Conclusion

In a representative sample of Swiss primary care patients, a substantial part shows multimorbidity with a high prevalence of chronic diseases, multiple drug treatment, and care dependency. These data may serve to be compared with other patient groups or other primary care system.

## Electronic supplementary material

Below is the link to the electronic supplementary material.

Supplementary material 1 (DOCX 434 kb)
