# Supplementary material for: Chronic conditions and multimorbidity in a primary care population: a study in the Swiss Sentinel Surveillance Network (Sentinella)
Source: Int J Public Health. 2018 May 21;63(9):1017–26. doi: 10.1007/s00038-018-1114-6 (PMC6245242; doi:10.1007/s00038-018-1114-6)
Supplement: Supplementary file 1 — Supplementary material 1 (DOCX 434 kb) [file 38_2018_1114_MOESM1_ESM.docx]

**Chronic Conditions and Multimorbidity in a Primary Care Population**

A Study in the Swiss Sentinel Surveillance Network (*Sentinella*)

*electronic supplementary materials*

**List of appendices to this paper**

**Appendix A: procedures for data collection in the practices**

**Appendix B: determining the denominator**

**Appendix C: frequently asked questions**

**Appendix D: strobe statement**

**Appendix E: e-tables and e-figures**

**Appendix A
Procedures for data collection in the practices (for paper and pencil patient records)**

The practice nurse informed the patients that we had to note study statistics. She asked the patient whether the list of chronically taken drugs was still up-to-date. If not, she made a note for the doctor. She asked the patients whether they were cared for by their family or by the community nurse. She also asked the patients whether they had been hospitalized during the preceding year.

She filled in the following fields on the questionnaire: last name, first name, gender, year of birth, hospitalization, care-dependency, number of chronic conditions and previous visits during the fortnight interval. She made a post-it note for every patient who had been seen during the study period.

She presented the patient files twice daily to the physician. He then controlled the fields that were coded by the nurse and encoded the fields “number of prescribed drugs taken regularly” and “Thurgau Morbidity Index” himself.

*This process required a coding time of 1.6 mins per patient for the nurse and 0.7 mins per patient for the physician (Gnädinger 2018, personal communication).*

**Appendix B**

**Determining the denominator**

For this study, you should transmit only the data of the patient file and not enter new data. If you cannot answer an item, mark “unknown” or “9/99” (Morbidity index, drug or condition number). Each field requires an entry, except “repeat visit”. Some information can be filled in by the practice nurse; but final checking and correcting before sending it to *Sentinella* administration is up to the physician.

*Care-dependency*

The possible answers are 1^[[1]](#footnote-1)^ = “yes, by proxies”, 2 = “yes, by community nurse”, 3 = “yes, by institution”, 4 = “none”, 5 = “unknown”. If a person makes more than one yes-answer, then select the higher number. For the study, people requiring home help or meal service are coded with “2”. Examples: Persons living in a home for the elderly are coded with “3”. People living in a residence for the elderly and visited by a community nurse are coded with “2”. People living at home and cared for by their family (i.e., children, demented) are coded with “1”.

*Number of drugs*

Each pharmacological preparation which is *chronically* prescribed scores with one point per active substance included. We consider a treatment to be chronic if it is applied for at least one month. Medication for shorter treatment periods (e.g. antibiotics) should not be included. Eye drops, inhalations or nasal sprays count only if a *systemic* effect is intended (calcitonine nasal spray) or must be accepted as unavoidable (timolol eye drops). We also want to register transdermal, subcutaneous or vaginal hormone delivering systems, or medication prescribed by a specialist (gynecologist: contraceptive pills). Herbal medicine counts – independent of the number of plant extracts contained – as one drug. Homeopathy such as Schüssler salts are not counted as mediaction. Multivitamins count only if taken for a medical reason (gastric bypass) and not if considered a tonic; they count as one drug. Oncologic treatment by a hospital also counts. Whether the patient applies the drug as scheduled or not, does not change her/his medication score. Medication on demand or self-medication is not counted. The qualifying date is the one of the recording. If you do not have assured data on medication, record “99”.

| **Table 1 Recording of prescribed drugs taken regularly**   \| *Does not count* \| Homeopathy, Schüssler salts, externals (without systemic effect), vitamins (if not medically indicated), on-demand medication, self-medication. Eye drops. Nasal spray without systemic effect. Short-time treatment (less than one month**).** \| \| --- \| --- \| \| *Counts as* ***one*** *drug* \| Herbal medicine, multivitamins (if medically indicated) \| \| *Counts per substance included* \| All other medication \| |
| --- | --- | --- | --- | --- | --- | --- |

Examples: Aclasta® (zoledronic acid) 5 mg once yearly i.v.: 1 point. Calcimagon D3® (cholecalciferol, calcium carbonas) twice daily: 2 points. Exforge HCT® (valsartan, amlodipine, hydrochlorothiazide) once daily: 3 points. Testogel® gel (testosterone) one daily transdermal application: 1 point. Excipial Lipolotio® two daily applications: 0 point. ReBalance® 500 mg (hypericum perforatum) once daily: 1 point. Ceres petasites D6 alcoholic drops, 5 drops trice daily: 0 point. Implanon® (etonogestrel), subcutaneous, for three years: 1 point. Miacalcic® nasal spray 200 μg twice daily: 1 point.

*Number of Conditions*

Each chronic condition receives 1 point, regardless of whether it is active (hypertension, actively treated), latent (elevated fasting blood glucose) or inactive (state after cholecystectomy). If the condition was important enough to be recorded in the patient file, it is important enough to count for this study! Exceptions: Drug allergies count as 1 point, even if they are multiple. An acute disease which is mainly chronic, but was not yet recorded as a chronic condition, should be counted for this study (e.g., activated knee osteoarthritis).

*Thurgau Morbidity Index*

You may note values from 0 to 6. Relevant for coding is the *worst* health state as caused by the chronic condition during the previous 12 months. For coding you follow the scheme from Figure 1. You start at the top. If the patient has at least **two severe conditions** (A), you code “6”. If the patient has **one severe** condition and at least **three mild to moderate** conditions, the code is “5”, if there are **less than three mild to moderate condition**, one codes “4” (B). If the patient has **no severe** condition but **at least three** mild to moderate ones, you encode “3”, and if the patient has only **one or two** mild to moderate conditions, the code is “2” (C). If the patient has **no** chronic condition, but **risk factors** or **findings** to be regularly monitored, the code is “1”. If none of the above mentioned situations is the case, the code is “0” (D, “healthy”). If your record is not sufficient to select a code, you assign “9” for unknown.

**
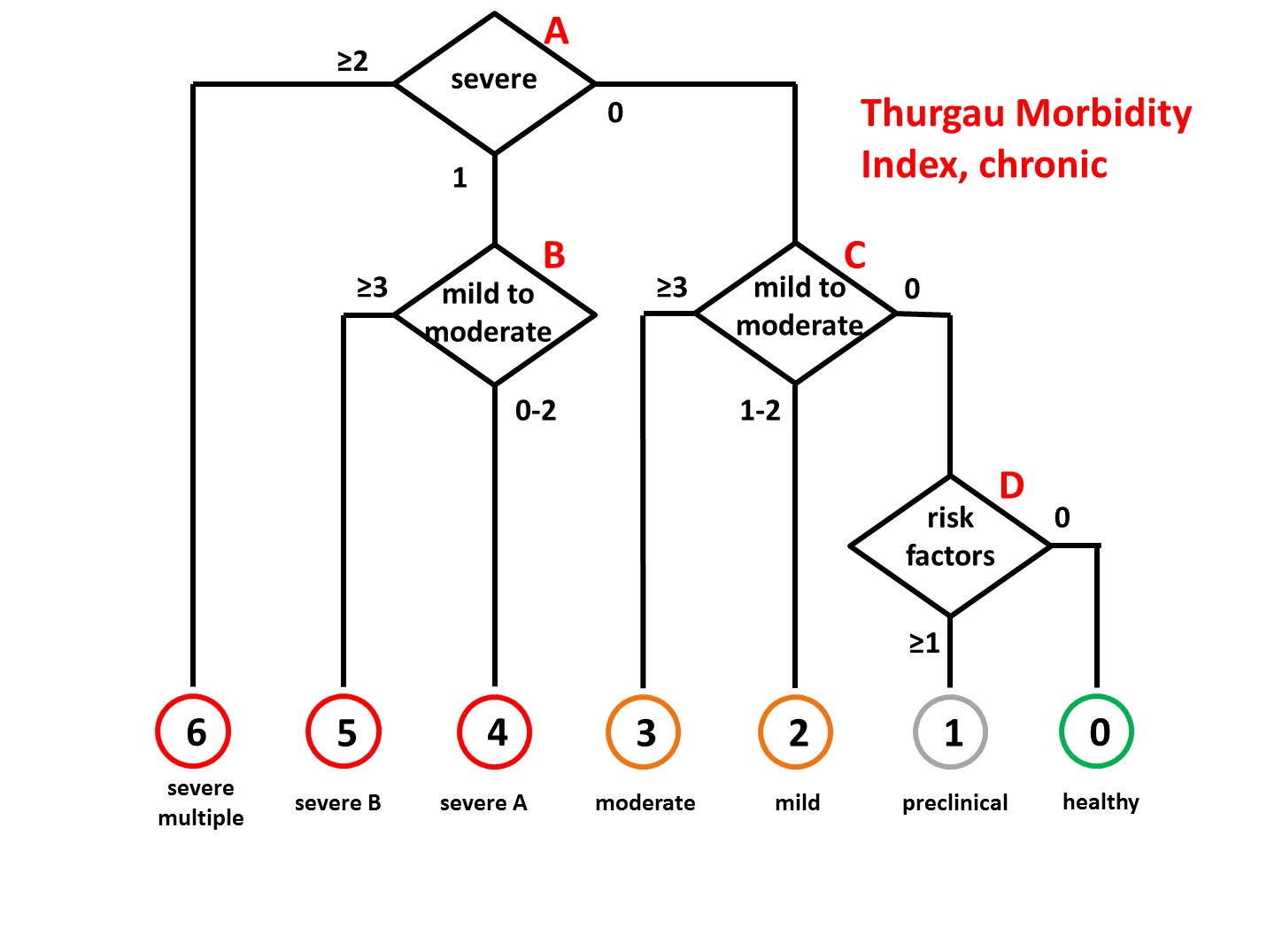
**

**Figure 1 Thurgau Morbidity Index (chronic scale)**

We consider a condition to be **severe** if there is:

- an active malignant tumor (non-melanoma skin cancer excluded)
- a chronic condition with instability, decompensation, acute thrust
- conditions with severely impaired organ function
- severe systemic disease
- rapidly progressive disease
- conditions with severe social impairment
- all other conditions which are considered to be severe

Cancer without relevant complaints is considered as mild to moderate (prostate). State after curative treatment of cancer is considered as “preclinical” or “healthy” (testicular). We consider the following conditions to be chronic: Primary chronic disease (osteoarthritis) or primary acute but not resolved after 12 months (hepatitis B).

Examples: A patient with currently compensated cardiac insufficiency was hospitalized three months earlier. His condition is considered to be severe. Rheumatoid arthritis treated with anti-TNF “biological treatment” without complaints counts to be mild to moderate. Osteoarthritis of the hip, operated six months before, scores for a severe condition, while the same patient, operated three years before and without complaints counts to be mild to moderate or preclinical. An obesity scores as mild to moderate, but if a gastric bypass operation had been performed during the previous 12 months, it counts to be severe. Addiction disease counts to be severe if stationary detoxification took place during the previous year or if its social consequences are severe. AIDS with a normalized immune system after medication is considered to be mild to moderate.

*Hospitalization*

If the patient had been hospitalized during the previous 12 months, you record “1”, if not “2”, if you do not know “3”. The cause of hospitalization does not matter for coding.

*Repeat visit*

If the patient is seen for a second or further time during the fortnight period, you should mark the field, even if she or he consults for a different disease than the first time. For first visits, you leave the field empty.

**Appendix C**

**Frequently asked questions (from:** [**www.medication-incidents.ch**](http://www.medication-incidents.ch)**)**

Q: The nurse confounded the medication of two residents in a home for the elderly. Do I have to fill in *two* notification forms?
**A: Yes, at least if you are in care of both residents.**

Q: During treatment with prednisolone and methotrexate, a patient had oral candidiasis. Later we learned that she had developed diabetes. Do I have to fill a form for that case?
**A: No, this was a monitoring error (since you did not control on time for diabetes); the drugs were applied in the normal way, and the undesired drug reaction (diabetes during corticosteroid treatment) is not to be noted in our study.**

Q: A patient was transferred from acute to geriatric care for continued i.v. antibiotic treatment. After three days, I was contacted by the family because the patient had not received his antibiotic treatment and was febrile. The i.v. antibiotics were not mentioned on the acute clinic transferring report. Do I have to report that case?
**A: No! For two reasons: Firstly, you were not in care of the patient when the incident happened, and secondly, our study aims to investigate incidents in primary care, but your patient was transferred from one secondary care unit to another.**

Q: Should I notify the daily problem of medication non-compliance of the patients?
**A: No, only if an incident arises out of it (e.g. overmedication when taking medication as planned).**

Q: Are children who are in a crèche to be considered as cared by an institution?
**A: No, only children who are in an institution for a condition (like cerebral palsy or schizophrenia) count as cared by the institution; normal children count as cared by the parents (when sick) or not cared by others.**

Q: Do we have to fill in all items in patients consulting a second time during denominator analysis?
**A: Yes, because we cannot assign the patients to their first visit by year of birth and gender alone.**

Q: Does the anti-rickets prophylaxis with vitamin D in healthy toddlers count as medicine?
**A: Yes.**

Q: Are prisoners supposed to be regarded as institutionalized patients?
**A: Yes, since usually they are not allowed to take their medication by themselves.**

**Appendix D**

STROBE Statement—Checklist of items that should be included in reports of ***cross-sectional studies***

|  | Item No | Recommendation |
| --- | --- | --- |
| **Title and abstract** | 1 | (*a*) Indicate the study’s design with a commonly used term in the title or the abstract **[yes]** |
|  |  | (*b*) Provide in the abstract an informative and balanced summary of what was done and what was found **[yes]** |
| Introduction | | |
| Background/rationale | 2 | Explain the scientific background and rationale for the investigation being reported **[yes]** |
| Objectives | 3 | State specific objectives, **[yes]** including any prespecified hypotheses **[no]** |
| Methods | | |
| Study design | 4 | Present key elements of study design early in the paper **[yes]** |
| Setting | 5 | Describe the setting, locations, and relevant dates, including periods of recruitment, exposure, follow-up, and data collection **[yes]** |
| Participants | 6 | (*a*) Give the eligibility criteria, and the sources and methods of selection of participants **[yes]** |
| Variables | 7 | Clearly define all outcomes, exposures, predictors, potential confounders, and effect modifiers. **[yes]** Give diagnostic criteria, if applicable **[no]** |
| Data sources/ measurement | 8* | For each variable of interest, give sources of data and details of methods of assessment (measurement). Describe comparability of assessment methods if there is more than one group **[yes]** |
| Bias | 9 | Describe any efforts to address potential sources of bias **[yes]** |
| Study size | 10 | Explain how the study size was arrived at **[yes]** |
| Quantitative variables | 11 | Explain how quantitative variables were handled in the analyses. If applicable, describe which groupings were chosen and why **[yes]** |
| Statistical methods | 12 | (*a*) Describe all statistical methods, including those used to control for confounding **[yes]** |
|  |  | (*b*) Describe any methods used to examine subgroups and interactions **[yes]** |
|  |  | (*c*) Explain how missing data were addressed **[yes]** |
|  |  | (*d*) If applicable, describe analytical methods taking account of sampling strategy **[no]** |
|  |  | (*e*) Describe any sensitivity analyses **[no]** |
| Results | | |
| Participants | 13* | (a) Report numbers of individuals at each stage of study—eg numbers potentially eligible, examined for eligibility, confirmed eligible, included in the study, completing follow-up, and analysed **[no]** |
|  |  | (b) Give reasons for non-participation at each stage **[yes]** |
|  |  | (c) Consider use of a flow diagram **[yes]** |
| Descriptive data | 14* | (a) Give characteristics of study participants (eg demographic, clinical, social) and information on exposures and potential confounders **[yes]** |
|  |  | (b) Indicate number of participants with missing data for each variable of interest **[yes]** |
| Outcome data | 15* | Report numbers of outcome events or summary measures **[no]** |
| Main results | 16 | (*a*) Give unadjusted estimates and, if applicable, confounder-adjusted estimates and their precision (eg, 95% confidence interval). Make clear which confounders were adjusted for and why they were included **[yes]** |
|  |  | (*b*) Report category boundaries when continuous variables were categorized **[yes]** |
|  |  | (*c*) If relevant, consider translating estimates of relative risk into absolute risk for a meaningful time period **[no]** |
| Other analyses | 17 | Report other analyses done—eg analyses of subgroups and interactions, and sensitivity analyses **[yes]** |
| Discussion | | |
| Key results | 18 | Summarise key results with reference to study objectives **[yes]** |
| Limitations | 19 | Discuss limitations of the study, taking into account sources of potential bias or imprecision. Discuss both direction and magnitude of any potential bias **[yes]** |
| Interpretation | 20 | Give a cautious overall interpretation of results considering objectives, limitations, multiplicity of analyses, results from similar studies, and other relevant evidence **[yes]** |
| Generalisability | 21 | Discuss the generalisability (external validity) of the study results **[yes]** |
| Other information | | |
| Funding | 22 | Give the source of funding and the role of the funders for the present study and, if applicable, for the original study on which the present article is based **[yes]** |

*Give information separately for exposed and unexposed groups.

**Note:** An Explanation and Elaboration article discusses each checklist item and gives methodological background and published examples of transparent reporting. The STROBE checklist is best used in conjunction with this article (freely available on the Web sites of PLoS Medicine at http://www.plosmedicine.org/, Annals of Internal Medicine at http://www.annals.org/, and Epidemiology at http://www.epidem.com/). Information on the STROBE Initiative is available at www.strobe-statement.org.

**Appendix E: e-Tables and e-Figures**

**List of contents**

*e-tables*

e1 Difficulties with morbidity item coding

e2 Morbidity indicators by age category and gender

e3 Correlation matrix (Spearman’s Rho)

*e-figures*

e1 Flow chart of the study and reporting rates

e2 Number of chronic conditions

e3 Evans Index value

e4 Mean Thurgau Morbidity Index values (CI 95%) by age and linguistic region

e5 Mean number of prescribed drugs taken regularly (CI 95%) by age and linguistic region

e6 Mean number of chronic conditions (CI 95%) by age and linguistic region

e7 Mean Evans’ Index (CI 95%) by age and linguistic region

e8 Frequencies of Thurgau Morbidity Index values in comparison with historical data

**Table e1. Difficulties with morbidity item coding**.

|  | Difficulty with morbidity item coding | | | |
| --- | --- | --- | --- | --- |
| Item | none | a little | important | very important |
| Previous hospitalization | 101 (78.3%) | 25 (19.4%) | 3 (2.3%) | 0 (0.0%) |
| Care-dependency | 91 (70.5%) | 31 (24.0%) | 6 (4.7%) | 1 (0.8%) |
| Medication count | 72 (55.8%) | 44 (34.1%) | 13 (10.1%) | 0 (0.0%) |
| Condition count | 56 (43.4%) | 51 (39.5%) | 22 (17.1%) | 0 (0.0%) |
| TMI | 52 (40.3%) | 59 (45.7%) | 17 (13.2%) | 1 (0.8%) |
| Repeat visit | 96 (74.4%) | 27 (20.9%) | 6 (4.7%) | 0 (0.0%) |

We received 145 questionnaires from 180 *Sentinella* physicians (response rate 80.6%, mostly there was only one questionnaire per practice). Amongst all physicians, 16 respondents did not participate in the morbidity study or answer the questions concerning coding difficulties. The *effort* to fill in the morbidity questionnaires was considered to be “manageable”: 61 (47.3%), “big effort”: 56 (43.4%), “too much effort”: 12 (9.4%), but no one “impossible”. The *time* to code all morbidity items for one patient was estimated by 113 respondents (79.6%): the mean value was 3.5±2.0 min.

Gnädinger (2018) Chronic Conditions and Multimorbidity in a Primary Care Population. A Study in the Swiss Sentinel Surveillance Network (*Sentinella*). International Journal of Public health

**Table e2 Age and gender proportion of patients, percent values (%), general practitioner data**

| Age groups, years | male patients | | female patients | |
| --- | --- | --- | --- | --- |
|  | *Sentinella*  n = 10’040 | NewIndex*  n = 5’282’285 | *Sentinella*  n = 11’878 | NewIndex*  n = 6‘898‘625 |
| 91 and over | 1.8 | 1.5 | 3.8 | 3.0 |
| 81 to 90 | 9.6 | 10.0 | 13.3 | 13.1 |
| 71 to 80 | 14.9 | 16.6 | 15.7 | 16.1 |
| 61 to 70 | 16.8 | 18.1 | 14.8 | 15.2 |
| 51 to 60 | 16.7 | 16.6 | 14.6 | 14.6 |
| 41 to 50 | 14.3 | 13.0 | 13.1 | 13.3 |
| 31 to 40 | 10.2 | 8.9 | 10.4 | 9.7 |
| 21 to 30 | 9.0 | 7.4 | 8.5 | 8.3 |
| 11 to 20 | 5.2 | 5.5 | 4.6 | 5.1 |
| 0 to 10 | 1.5 | 2.4 | 1.2 | 1.6 |

* NewIndex data correspond to health (but *not* accident) insurance tariff, consultations and home visits, for the year 2014, n=12’180’910, 56.6% females. *Sentinella* data correspond to 21’918 consultation or home visit records (54.2% female patients). Median testing revealed that the male patient group of NewIndex was slightly older (p<0.001, median estimation by Hampel: *Sentinella* 55.2 years and NewIndex 56.3 years).

Gnädinger (2018) Chronic Conditions and Multimorbidity in a Primary Care Population. A Study in the Swiss Sentinel Surveillance Network (*Sentinella*). International Journal of Public health

**Table e3 Age and gender proportion of patients, percent values (%), pediatricians**

| Age groups, years | male patients | | female patients | |
| --- | --- | --- | --- | --- |
|  | *Sentinella*  n = 2’566 | NewIndex*  n = 857’850 | *Sentinella*  n = 2’331 | NewIndex*  n = 767’058 |
| 20 and over | 0.9 | 1.5 | 0.8 | 3.0 |
| 16 to 20 | 5.5 | 4.0 | 6.1 | 5.0 |
| 11 to 15 | 16.4 | 17.9 | 16.1 | 18.4 |
| 5 to 10 | 30.6 | 26.9 | 28.7 | 26.1 |
| 4 | 6.4 | 7.4 | 6.7 | 7.2 |
| 3 | 7.0 | 8.1 | 7.0 | 7.7 |
| 2 | 11.1 | 10.4 | 12.0 | 9.9 |
| 0 to 1 | 22.1 | 23.8 | 22.6 | 22.7 |

* NewIndex data correspond to health (but *not* accident) insurance tariff, consultations and home visits, whole year 2014, n=1’624’908, 47.2% females. *Sentinella* data correspond to 4’897 consultation or home visit records (47.6% female patients). Median testing revealed that the female patient group of NewIndex was slightly older (p<0.001, median estimation by Hampel: *Sentinella* 4.8 years and NewIndex 5.3 years).

Gnädinger (2018) Chronic Conditions and Multimorbidity in a Primary Care Population. A Study in the Swiss Sentinel Surveillance Network (*Sentinella*). International Journal of Public health

**Table e4. Morbidity indicators by age category and gender**

| **Age categories** | **Hospitalisation (percent)** | | **Care-dependency* (percent)** | | **Conditions (Median/IQR)** | | **Drugs (Median/IQR)** | | **Evans’ Index (Median/IQR)** | | **TMI**  **(Median/IQR)** | | **Repeat visit (percent)** | |
| --- | --- | --- | --- | --- | --- | --- | --- | --- | --- | --- | --- | --- | --- | --- |
|  | *male* | *female* | *male* | *female* | *male* | *female* | *male* | *female* | *male* | *female* | *male* | *female* | *male* | *female* |
| **0 - 10** | 7.2 | 8.3 | n.a. | n.a. | 0 (0-0) | 0 (0-0) | 0 (0-1) | 0 (0-1) | 0 (0-1) | 0 (0-1) | 0 (0-0) | 0 (0-0) | 5.2 | 4.7 |
| **11 - 20** | 3.8 | 5.2 | n.a. | n.a. | 0 (0-1) | 0 (0-1) | 0 (0-0) | 0 (0-0) | 0 (0-1) | 0 (0-1) | 0 (0-1) | 0 (0-1) | 6.1 | 6.1 |
| **21 - 30** | 6.0 | 8.9 | 3.6 | 2.8 | 0 (0-1) | 1 (0-1) | 0 (0-0) | 0 (0-1) | 0 (0-2) | 1 (0-2) | 0 (0-1) | 0 (0-1) | 8.4 | 8.3 |
| **31 - 40** | 9.6 | 9.4 | 3.7 | 2.7 | 1 (0-2) | 1 (0-2) | 0 (0-1) | 0 (0-1) | 1 (0-3) | 1 (0-4) | 1 (0-2) | 1(0-2) | 7.6 | 8.4 |
| **41 - 50** | 11.5 | 9.4 | 5.2 | 3.7 | 1 (0-3) | 1 (0-3) | 1 (0-2) | 1 (0-2) | 2 (0-5) | 3 (1-5) | 1 (0-2) | 1 (0-2) | 8.6 | 7.4 |
| **51 - 60** | 13.8 | 14.3 | 5.5 | 6.0 | 2 (1-4) | 2 (1-4) | 2 (0-4) | 2 (0-4) | 4 (2-7) | 4 (2-7) | 2 (1-3) | 2 (1-3) | 8.3 | 7.5 |
| **61 - 70** | 19.6 | 16.0 | 6.4 | 9.7 | 3 (2-4) | 3 (2-5) | 3 (1-5) | 3 (1-5) | 6 (3-9) | 6 (4-10) | 3 (1-3) | 2 (1-3) | 7.8 | 8.0 |
| **71 - 80** | 27.3 | 22.9 | 17.7 | 18.0 | 4 (2-6) | 4 (2-5) | 4 (3-7) | 4 (2-6) | 8 (5-12) | 8 (5-12) | 3 (2-4) | 3 (2-4) | 7.1 | 8.1 |
| **81 - 90** | 31.2 | 30.9 | 38.2 | 49.8 | 4 (3-7) | 4 (3-6) | 5 (3-7) | 5 (4-8) | 10 (7-14) | 10 (7-14) | 4 (3-5) | 3 (3-5) | 10.8 | 9.1 |
| **91 and over** | 31.7 | 32.0 | 79.0 | 74.7 | 5 (3-6) | 4 (3-6) | 5 (3-7) | 5 (3-8) | 10 (7-13) | 9 (6-14) | 4 (3-5) | 4 (3-5) | 8.0 | 8.7 |

***** Because of question ambiguity, the care-dependency category “care by parents / proxies” could not be evaluated in children and teenagers, so age categories 1 and 2 are without data.

Gnädinger (2018) Chronic Conditions and Multimorbidity in a Primary Care Population. A Study in the Swiss Sentinel Surveillance Network (*Sentinella*). International Journal of Public health

**Table e5. Correlation matrix of the study variables (Spearman’s Rho)**

|  |  | Gender | Patient's age | Hospitalization in previous year | Care-dependency | Number of prescribed drugs regularly taken | Number of chronic conditions | Thurgau Morbidity Index (TMI) | Evans' Index |
| --- | --- | --- | --- | --- | --- | --- | --- | --- | --- |
| Gender | correlation coefficient | 1.000 | .072^**^ | .008 | .062^**^ | .070^**^ | .058^**^ | .051^**^ | .065^**^ |
|  | Sig. (2-sided) |  | .000 | .188 | .000 | .000 | .000 | .000 | .000 |
|  | N | 26815 | 26803 | 24749 | 20689 | 24413 | 24449 | 24407 | 24324 |
| Patient's age | correlation coefficient | .072^**^ | 1.000 | .219^**^ | .356^**^ | .692^**^ | .719^**^ | .693^**^ | .725^**^ |
|  | Sig. (2-sided) | .000 |  | .000 | 0.000 | 0.000 | 0.000 | 0.000 | 0.000 |
|  | N | 26803 | 26816 | 24752 | 20694 | 24419 | 24456 | 24412 | 24331 |
| Hospitalization in previous year | correlation coefficient | .008 | .219^**^ | 1.000 | .295^**^ | .291^**^ | .281^**^ | .337^**^ | .299^**^ |
|  | Sig. (2-sided) | .188 | .000 |  | 0.000 | 0.000 | 0.000 | 0.000 | 0.000 |
|  | N | 24749 | 24752 | 24761 | 20544 | 23723 | 23874 | 23998 | 23718 |
| Care-dependency | correlation coefficient | .062^**^ | .356^**^ | .295^**^ | 1.000 | .350^**^ | .308^**^ | .397^**^ | .342^**^ |
|  | Sig. (2-sided) | .000 | 0.000 | 0.000 |  | 0.000 | 0.000 | 0.000 | 0.000 |
|  | N | 20689 | 20694 | 20544 | 20701 | 20175 | 20204 | 20156 | 20213 |
| Number of prescribed drugs regularly taken | correlation coefficient | .070^**^ | .692^**^ | .291^**^ | .350^**^ | 1.000 | .817^**^ | .781^**^ | .936^**^ |
|  | Sig. (2-sided) | .000 | 0.000 | 0.000 | 0.000 |  | 0.000 | 0.000 | 0.000 |
|  | N | 24413 | 24419 | 23723 | 20175 | 24424 | 24255 | 23861 | 24272 |
| Number of chronic conditions | correlation coefficient | .058^**^ | .719^**^ | .281^**^ | .308^**^ | .817^**^ | 1.000 | .830^**^ | .957^**^ |
|  | Sig. (2-sided) | .000 | 0.000 | 0.000 | 0.000 | 0.000 |  | 0.000 | 0.000 |
|  | N | 24449 | 24456 | 23874 | 20204 | 24255 | 24461 | 23996 | 24282 |
| Thurgau Morbidity Index (TMI) | correlation coefficient | .051^**^ | .693^**^ | .337^**^ | .397^**^ | .781^**^ | .830^**^ | 1.000 | .840^**^ |
|  | Sig. (2-sided) | .000 | 0.000 | 0.000 | 0.000 | 0.000 | 0.000 |  | 0.000 |
|  | N | 24407 | 24412 | 23998 | 20156 | 23861 | 23996 | 24420 | 23864 |
| Evans' index | correlation coefficient | .065^**^ | .725^**^ | .299^**^ | .342^**^ | .936^**^ | .957^**^ | .840^**^ | 1.000 |
|  | Sig. (2-sided) | .000 | 0.000 | 0.000 | 0.000 | 0.000 | 0.000 | 0.000 |  |
|  | N | 24324 | 24331 | 23718 | 20213 | 24272 | 24282 | 23864 | 24336 |

** The correlation is significant at the level of p=0.01 (2-sided).

Gnädinger (2018) Chronic Conditions and Multimorbidity in a Primary Care Population. A Study in the Swiss Sentinel Surveillance Network (*Sentinella*). International Journal of Public health


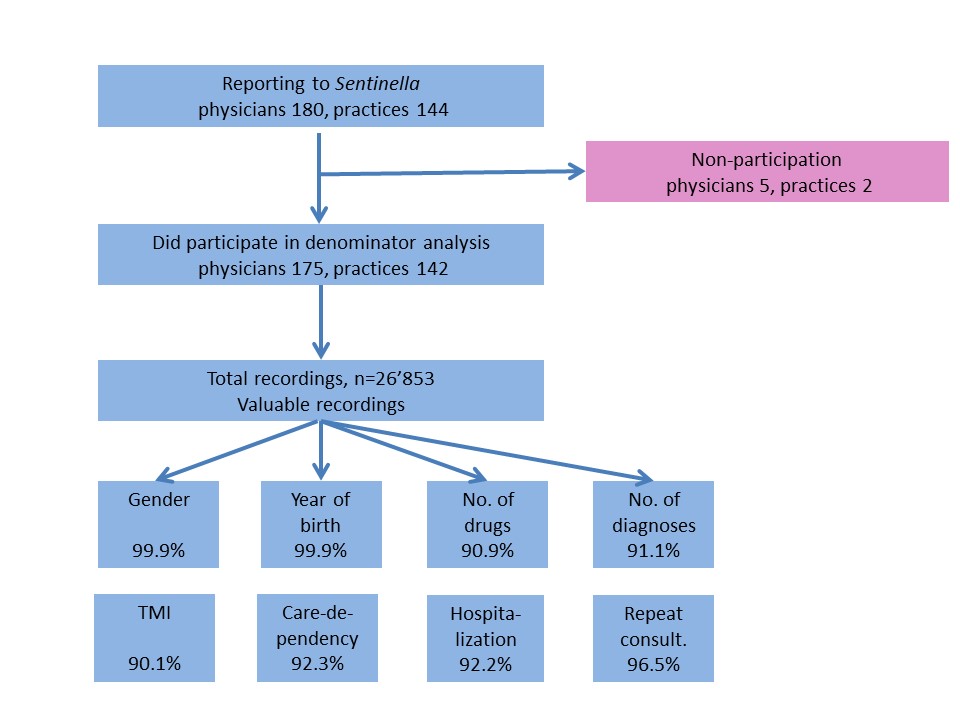


**Figure e1 Flow chart of the study and reporting rates**Care-dependency was only evaluated for adults >19 years. The number of observations without missing values was 18’297 (only adult patients).

Gnädinger (2018) Chronic Conditions and Multimorbidity in a Primary Care Population. A Study in the Swiss Sentinel Surveillance Network (*Sentinella*). International Journal of Public health


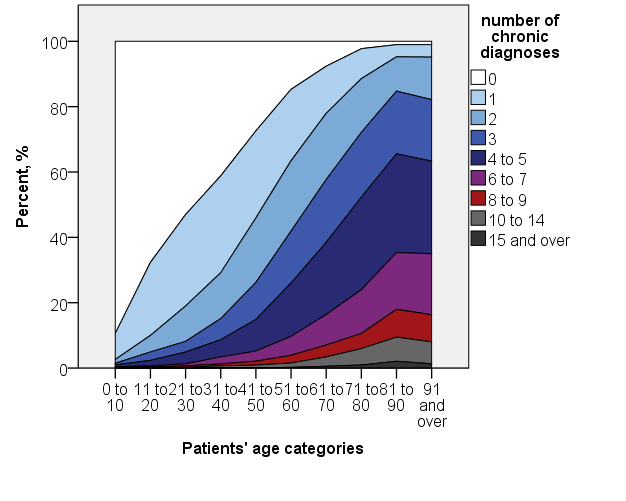


**Figure e2. Number of chronic conditions, percent (%).** Graduations denote the entire class.

Gnädinger (2018) Chronic Conditions and Multimorbidity in a Primary Care Population. A Study in the Swiss Sentinel Surveillance Network (*Sentinella*). International Journal of Public health


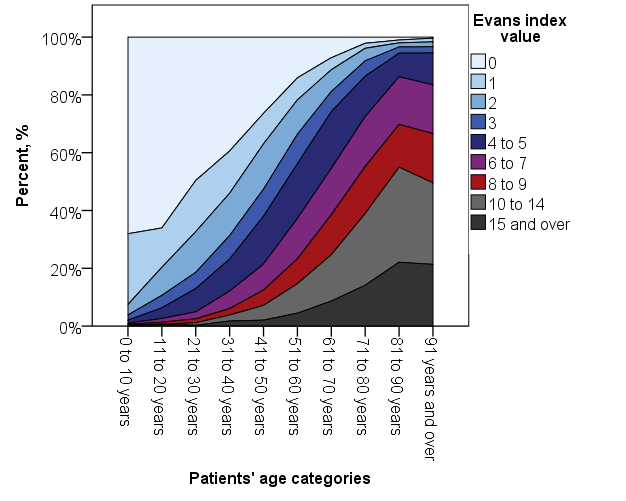


**Figure e3. Evans’ Index value, percent.** Graduations denote the entire class.

Gnädinger (2018) Chronic Conditions and Multimorbidity in a Primary Care Population. A Study in the Swiss Sentinel Surveillance Network (*Sentinella*). International Journal of Public health


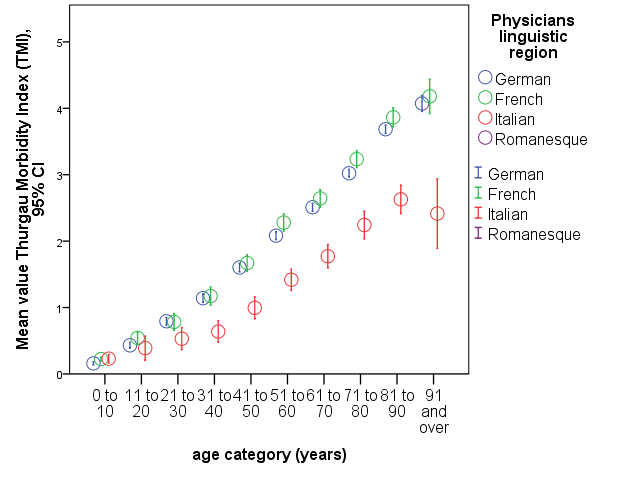


**Figure e4. Mean Thurgau Morbidity index values (CI 95%) by age and linguistic region**

Gnädinger (2018) Chronic Conditions and Multimorbidity in a Primary Care Population. A Study in the Swiss Sentinel Surveillance Network (*Sentinella*). International Journal of Public health


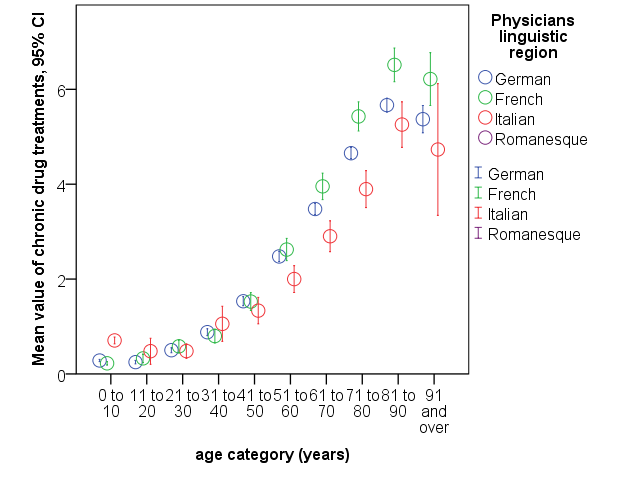
 **Figure e5. Mean number of prescribed drugs taken regularly (CI 95%) by age and linguistic region**

Gnädinger (2018) Chronic Conditions and Multimorbidity in a Primary Care Population. A Study in the Swiss Sentinel Surveillance Network (*Sentinella*). International Journal of Public health


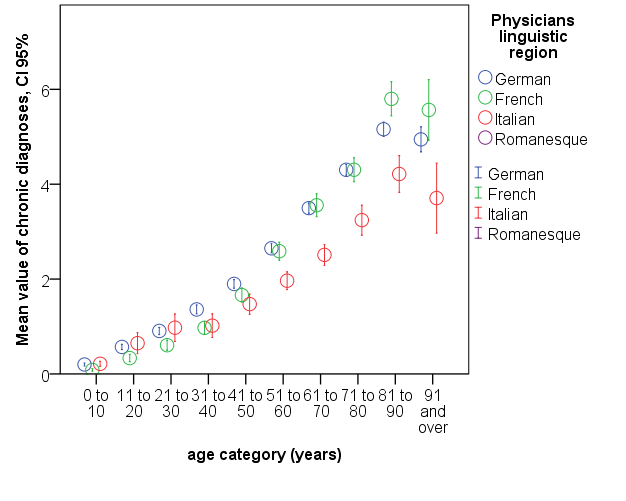
**Figure e6. Mean number of chronic conditions (CI 95%) by age and linguistic region**

Gnädinger (2018) Chronic Conditions and Multimorbidity in a Primary Care Population. A Study in the Swiss Sentinel Surveillance Network (*Sentinella*). International Journal of Public health


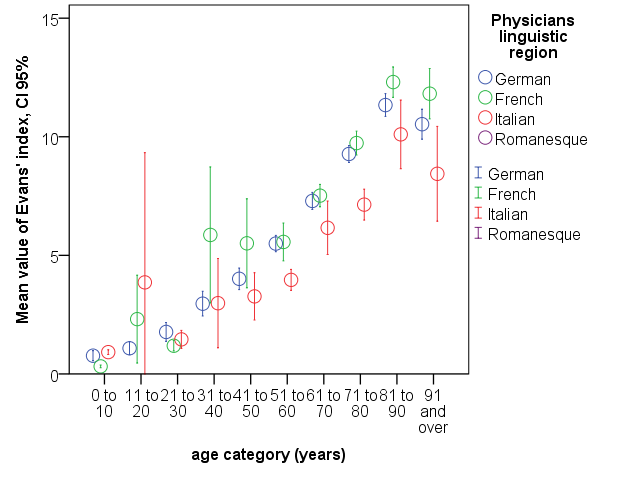


**Figure e7. Mean Evan’s Index (CI95%) by age and linguistic region**

Gnädinger (2018) Chronic Conditions and Multimorbidity in a Primary Care Population. A Study in the Swiss Sentinel Surveillance Network (*Sentinella*). International Journal of Public health


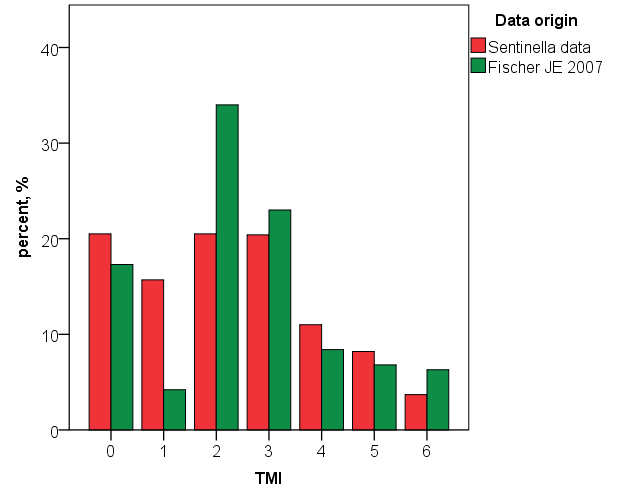


**Figure e8. Frequencies of Thurgau Morbidity Index values in comparison with historical data (Fischer 2007) (all age groups and both genders, GPs only).** Fischer et al. reported an intra-class correlation coefficient of 0.72 in trained physicians. In contrast to the data by Fischer et al. we found lower proportions of codes 2, 3 and 6 and more codes 0 and 1. However, the latter study did not include consecutive patients but rather a “convenience sample” preferring higher TMI codes for analyzing healthcare cost, i.e. the smaller groups of more severely diseased patients (personal communication of Joachim E. Fischer, Mannheim).

Gnädinger (2018) Chronic Conditions and Multimorbidity in a Primary Care Population. A Study in the Swiss Sentinel Surveillance Network (*Sentinella*). International Journal of Public health

1. This issue has led to some ambiguity in our study. In a new study, we recommend to include the new category “minors cared by parents”. [↑](#footnote-ref-1)
